# Identifying climate drivers of infectious disease dynamics: recent advances and challenges ahead

**DOI:** 10.1098/rspb.2017.0901

**Published:** 2017-08-16

**Authors:** C. Jessica E. Metcalf, Katharine S. Walter, Amy Wesolowski, Caroline O. Buckee, Elena Shevliakova, Andrew J. Tatem, William R. Boos, Daniel M. Weinberger, Virginia E. Pitzer

**Affiliations:** 1 Department of Ecology and Evolutionary Biology, Princeton University, Princeton, NJ, USA; 2 Office of Population Research, Woodrow Wilson School, Princeton University, Princeton, NJ, USA; 3 Department of Epidemiology of Microbial Diseases, Yale School of Public Health, Yale University, New Haven, CT, USA; 4 Department of Geology and Geophysics, Yale University, New Haven, CT, USA; 5 Department of Epidemiology, Johns Hopkins Bloomberg School of Public Helath, Baltimore, MD, USA; 6 Department of Epidemiology, Harvard T.H. Chan School of Public Health, Boston, MA, USA; 7 Center for Communicable Disease Dynamics, Harvard T.H. Chan School of Public Health, Boston, MA, USA; 8 NOAA/GFDL, Princeton, NJ, USA; 9 Flowminder Foundation, Stockholm, Sweden; 10 WorldPop project, Department of Geography and Environment, University of Southampton, Southampton, UK

**Keywords:** climate, infection, statistical model, mathematical model, mechanism, climate change

## Abstract

Climate change is likely to profoundly modulate the burden of infectious diseases. However, attributing health impacts to a changing climate requires being able to associate changes in infectious disease incidence with the potentially complex influences of climate. This aim is further complicated by nonlinear feedbacks inherent in the dynamics of many infections, driven by the processes of immunity and transmission. Here, we detail the mechanisms by which climate drivers can shape infectious disease incidence, from direct effects on vector life history to indirect effects on human susceptibility, and detail the scope of variation available with which to probe these mechanisms. We review approaches used to evaluate and quantify associations between climate and infectious disease incidence, discuss the array of data available to tackle this question, and detail remaining challenges in understanding the implications of climate change for infectious disease incidence. We point to areas where synthesis between approaches used in climate science and infectious disease biology provide potential for progress.

## 1. Introduction

The role of environmental variables and climatic conditions in shaping human health has been recognized for centuries. Infectious diseases, in particular, may be sensitive to climatic conditions through their effects on abundance of vectors such as mosquitoes and ticks [1], pathogen survival outside of the host [2], environmental contamination and exposure to water-borne infections [3], dampening of host immunity [4], disruptions of health status associated with malnutrition linked to droughts or floods, and disruption of health systems by disasters such as floods or hurricanes. Consequently, shifts in climate—the average state of the atmosphere–ocean–land system over time, as well as the day-to-day variability of weather—may affect the burden of infectious diseases now and in the future [5].

Appropriately attributing changes in the burden of infectious diseases to climatic variables, and quantifying this relationship, is a necessary step in evaluating the potential impact of climate change [6]. However, this is complicated by a number of factors. One issue is that the available data for many pathogens often consist of only human cases of incident disease (rather than prevalence of infection in vectors and/or mild or asymptomatic hosts) and rarely span time horizons reflective of major climatic shifts. As a result, inference into the consequences of climate change requires building on core biological knowledge (e.g. experiments indicating vector or pathogen survival across the range of a specific climatic variable [7]), leveraging spatial variation and range limits associated with climatic conditions [8], or building mechanistic understanding from shorter time-series of climatic variables and disease incidence—a process which itself often builds on biological knowledge and/or spatial variation. Another issue is that while climate acts as an extrinsic driver, infectious disease dynamics also have intrinsic drivers, particularly fluctuations in population-level immunity and susceptibility, as well as the dynamics of human behaviour (e.g. increasing population size and mobility can modify previous geographical limits for vector-borne infections [9]). Disentangling these drivers requires careful statistical and model-based partitioning of possible links between climate and infectious disease incidence while accounting for features of host–pathogen biology such as asymptomatic carriage [10].

In order to attribute a change in infectious disease incidence to climate, Rogers & Randolph [1] specify three criteria: the change in infectious disease incidence must have occurred at the right time, in the right place and in the right direction (consistent with the hypothesized climate–disease relationship). The third criterion requires understanding the mechanism(s) by which climate may affect infectious disease incidence, while the former two criteria require careful analysis of spatio-temporal data. Here, we review the mechanisms by which climatic variables might affect infectious disease transmission, discuss challenges involved in linking climate drivers to infectious disease transmission, provide an overview of statistical and mechanistic models that can be used to quantify these connections, and discuss how these might contribute to generating future projections of the effects of climate on health. Previous reviews have outlined mechanisms underlying climate and infectious disease associations in non-human pathogens [11,12], reviewed conceptual challenges in detection and attribution [6], and provided an overview of core knowledge gaps [13]. Our focus is on infectious diseases in humans, and we draw from examples across a range of pathogens to concretely illustrate the methodological challenges and approaches that have been developed to grapple with projecting future infectious disease incidence under climate change.

## 2. Potential mechanisms linking climate and infectious diseases

Associations between climatic conditions and infectious disease incidence may be observed at a range of spatial and temporal scales, but associations alone do not indicate causal links. Establishing causality requires identifying whether the association is consistent with a hypothesized mechanism. There are a wealth of potential mechanisms linking climate and infectious diseases, which differ across ecological aspects of the human–pathogen interaction, including route of transmission.

Infectious diseases may be directly transmitted via airborne particles or fomites (e.g. influenza), or indirectly transmitted via food, water (e.g. cholera) or a vector (e.g. malaria, dengue), and could potentially involve non-human reservoir species (zoonotic pathogens, e.g. hantavirus). Each transmission route may be associated with different climatic drivers, outlined in the electronic supplementary material, table S1. The consequences of climatic variation can range from complete prevention of transmission (across geographical ranges or at certain times of the year) to shifting the magnitude of transmission.

The most direct approach to identifying a mechanistic impact of climate on infectious diseases is experimentation. However, pathogens for which good animal models of transmission exist (e.g. guinea pigs for influenza [2]), or for which experimental studies of vector dynamics can be deployed (e.g. *Aedes aegypti* for dengue [7]), remain a minority. Sometimes, general aspects of the thermal or broader environmental niche are known and can be used to inform the expected direction of the climate–disease relationship.

To appropriately attribute the impact of climate change on infectious disease incidence, we must move beyond simply identifying the direction of the climate–disease relationship to actually quantifying these relationships under natural conditions. Model-based approaches can link changes in climate variables to changes in disease incidence, ideally following intermediate steps on the hypothesized causal pathway. For instance, if rainfall and flooding are thought to influence the risk of exposure for a water-borne disease (e.g. typhoid fever) through increased contamination of drinking water, ideally one would link the hydrologically relevant climate variables to the prevalence of bacterial contamination of water supplies [14] prior to examining correlations with human incidence data.

Importantly, climate probably mediates infectious disease risk via multiple mechanisms, and variability in climatic variables may be even more important than mean levels [15–17]. These factors complicate identifying the role played by climatic variables in shaping the burden of infectious diseases. The scope of climatic variation that can be used to drive inference around the possible impacts of climate is an essential component of meeting this challenge, which we detail next, before outlining available methodological approaches.

## 3. Spatio-temporal scales of variation and confounding factors

Once a hypothesized mechanism is established, the next two criteria require determining whether changes in climate variables can be linked to changes in disease incidence in space or time. Several scales and types of spatio-temporal variation can be leveraged.

### (a) Spatial variation

The geographical range limits of infections can powerfully indicate the effects of climate. For many vector-borne infections, climatic changes (particularly increasing temperatures) could increase the geographical range of vectors and thereby increase the size of the at-risk population; conversely, decreases in geographical range could be projected if conditions become too dry [18]. Such changes have been modelled for infections like malaria [19,20], dengue [21], onchocerciasis [22], Chagas disease [23], West Nile virus [24], chikungunya [25] and Rift Valley fever virus [26], and vectors like sand flies [27] and black-legged ticks [28]. However, it is important (and often difficult) to differentiate between places that cannot support ongoing transmission of the pathogen due to suboptimal climatic conditions versus locations where the pathogen has yet to be introduced or has recently been eliminated by anthropogenic rather than climatic influences [29,30]. Control efforts are likely to be focused in areas of intense transmission, potentially obscuring key climatic conditions.

### (b) Seasonality

Seasonal variation is another powerful focus for disentangling climate–infectious disease relationships, providing a repeatable probe of the association between climatic drivers and transmission. Between-year variation, from small deviations [31] through to unusual climatic conditions [32], can be used to identify core climatic drivers if appropriate methods are deployed (see below). However, given the large number of candidate climatic variables, many of which also exhibit seasonal increases and decreases, spurious associations between climatic drivers and a focal infection are inevitable. Confounders linked to human behaviour or demography [33], or seasonality in immune function (e.g. associated with vitamin D metabolism [34]), further complicate inference (electronic supplementary material, S1). Combining seasonal variations with biological knowledge (e.g. [2]) and/or variation through space [35] can help to identify a signature of a driving covariate among multiple covarying climatic variables.

### (c) Multi-annual variation

Over longer time-courses, multi-annual fluctuations in climate, such as the El Niño Southern Oscillation (ENSO), provide strong signatures against which to test associations between climate and infectious diseases [36], and can help tease apart the effect of different climatic factors that do not necessarily covary on longer than seasonal time scales [37]. Non-stationary changes in climate (e.g. warming, multi-decadal drought) can also be examined, but establishing robust links with these longer-time-scale variations in climate requires amassing infectious disease time series of sufficiently long duration, which is challenging [38]. Even if data are available, long-term signatures of associations between climate and infectious disease dynamics may be overwhelmed by changes in surveillance practices and/or the introduction of interventions, as well as non-stationary changes in human ecology (e.g. increasing density, urbanization [29]) and evolution in pathogen (e.g. emergence of drug resistance [39]) or vector populations (e.g. climate-imposed selection may alter mosquito diapause, shifting the range of mosquito-borne pathogens [40]). Thus, observed associations between long-term changes in climate and infectious disease burden must be interpreted with caution, taking into account the underlying changes in the human and pathogen populations and their interface [30].

### (d) Combining spatial and temporal scales

‘Location-specific’ associations, which cannot be extrapolated to regions where the climate may differ, or temporal associations that do not align with range limits linked to climate, pose a serious challenge to characterizing the effects of climate change. In many instances, these may be indicative of non-mechanistic associations. However, it is also possible that the important drivers of transmission vary depending on the range of climatic conditions in a given location. For example, the climatic drivers of influenza transmission, or the shape of the climate–disease relationship, may vary between temperate and tropical regions [41,42]. Understanding how climate change might affect this pathogen will require accounting for these differences.

## 4. Estimating climate effects on infectious diseases

**Figure 1.**
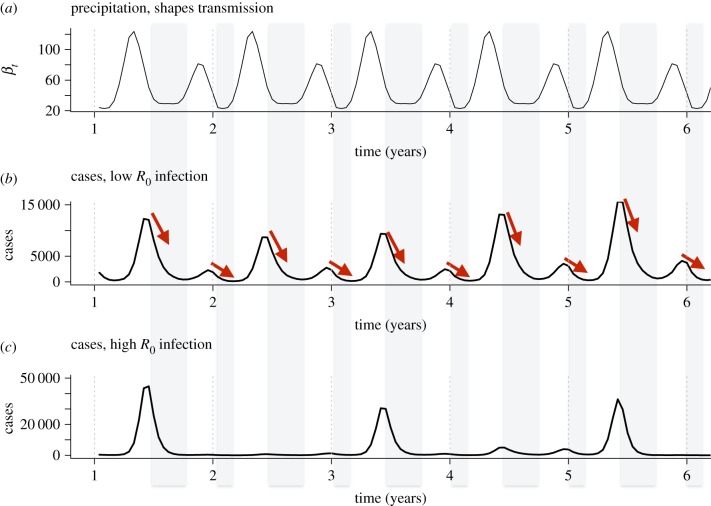
Issues with linking incidence and climate drivers for immunizing infections (*a*) Simulated precipitation over 6 years, set to reflect bimodal peaks in each year as observed (e.g. in Kenya), with periods of low precipitation shown in grey. (*b*) Resulting cases for a completely immunizing infection with a two-week generation time and a low basic reproductive number (*R*_0_) (set to *R*_0_ = 5) simulated using an SIR transmission dynamic model as in [43]. The resulting time series shows a clear footprint where periods of low precipitation and thus low transmission correspond to outbreaks turning over (red arrows), where conversely high precipitation is associated with increases in incidence. (*c*) Resulting cases for an identical infection simulated using an SIR model, but with a high basic reproductive number (*R*_0_ = 32). In this case, the resulting time series shows a much more erratic picture, with little direct indication of the impact of precipitation on cases, especially in low incidence years (i.e. no increase in cases with increases in precipitation in year 2), as a result of the dominant multi-annual period resulting from the intrinsic dynamics.

The scales of variation delineated have the potential to inform our estimates of the association between climate drivers and disease incidence. However, for communicable diseases (pathogens transmitted from person to person, either directly or indirectly), several factors make this challenging. First, the infectious process is typically unobserved. Second, many infections are to some degree immunizing, meaning that one new infection both magnifies transmission, but also depletes the pool of susceptible individuals, resulting in feedbacks in the transmission dynamics which can obscure climate signatures (figure 1). Attributing changes in infectious diseases to specific climate drivers thus requires accounting for nonlinearities in the risk of exposure and susceptible depletion, a fact recognized since at least the 1950s, when Hope Simpson quantified ‘infectiousness’ for measles, chickenpox and mumps by carefully accounting for individuals in households who had previously been exposed and were thus no longer susceptible. He found no seasonal variation in ‘infectiousness’, despite seasonality in incidence [44]. The core of his analysis underscores the essential limitation of using traditional time-series approaches for infectious disease dynamics—the population exposed and at risk changes rapidly as a result of the very dynamics of infection. Autocorrelations and lags that do not take into account the underlying biology can result in biases [45] and more complex inferential frameworks may be required.

**Figure 2.**
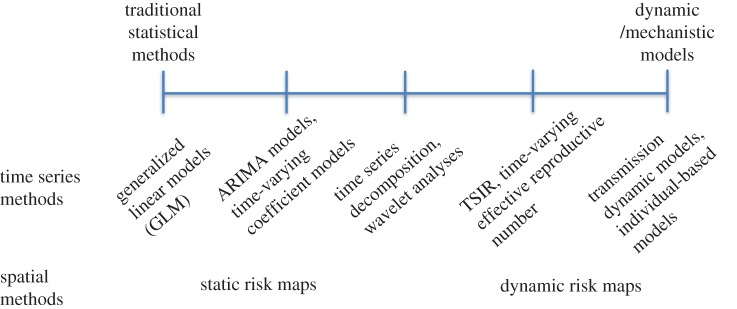
Schematic of the range of modelling approaches ordered across a spectrum of increasing incorporation of mechanism. (Online version in colour.)

An array of methods is available for disentangling links between climate and infectious diseases, ranging from statistical to mechanistic (i.e. dynamic) modelling approaches (figure 2). Statistical models focus on matching patterns of variation in climatic variables with the distribution of observed cases of disease in time and/or space; mechanistic models seek to dissect these relationships by explicitly accounting for the processes of transmission of infection and observation of disease [1]. Both approaches are useful, but their applicability depends on characteristics of the pathogen and the host–pathogen relationship (see the electronic supplementary material, table S2.)

### (a) Traditional time-series modelling approaches

Statistical modelling approaches for climate–disease associations include regression approaches, such as generalized linear models or generalized additive models (which can also include time-dependent components), and conventional time-series models, such as autoregressive integrated moving average (ARIMA) models. Their appropriateness is shaped by the degree to which hidden processes linked to transmission or immunity result in confounding or non-independence (electronic supplementary material, table S2). Where climate affects susceptibility to symptomatic disease (in turn affecting detectability by the surveillance system) rather than the infection process, as reported for *Streptococcus pyogenes* [46], nonlinear feedbacks between climate and disease incidence should be minimal, and statistical approaches appropriate. Likewise, if the primary reservoir for infection is a non-human host and the impact of climate is to mediate pathogen exposure, possibly by affecting vectors, then nonlinear modelling approaches are not required unless relevant dynamic feedbacks are identified. Accordingly, traditional time-series modelling approaches have been applied to examine associations between extreme precipitation and water-borne diseases in the USA [47], and rainfall, humidity and cutaneous leishmaniasis in Brazil [48]. For infections with relatively low transmission rates, and a long generation interval, traditional time-series approaches could also be applied, because there will only be weak dependence between consecutive observations (i.e. incident cases of disease; figure 1). Finally, conventional time-series approaches are appropriate for exploratory analyses to identify potentially relevant variables for further inquiry using mechanistic approaches [35].

When carefully trained, evaluated and tested (e.g. via out-of-sample prediction), non-mechanistic autoregressive models may have good predictive power for forecasting burden over shorter time horizons [49], even outperforming models that incorporate the ‘true’ underlying drivers; but over the longer term, non-stationary changes in climate, or variables like demography and susceptible depletion, may complicate this picture.

### (b) Time-series decomposition, wavelet analysis and synchrony

The periodicity in the dynamics of some infectious diseases as well as climate (including both seasonal and multi-annual cycles) lends itself to a range of methods specifically designed to quantify non-stationary associations between time series, such as changes in mean, variance and/or period of oscillations over time. Methods for time-series decomposition (including moving averages, seasonal and trend decomposition using Loess and Bayesian structural time-series models [50]) seek to separate out long-term trends in incidence from seasonal and multi-annual cycles and random ‘noise’, bridging the gap between traditional statistical approaches and mechanistic models. Wavelet analysis is especially appropriate for identifying changes in the periodicity and/or phase of incident cases and correlating them with exogenous variables [51]. Wavelets have been used to evaluate the synchrony between rainfall and cholera across Africa [52] and Haiti [53], and to suggest that warmer locations serve as sources of out-going waves of dengue across Southeast Asia [54]. Such approaches may be particularly powerful in settings where nonlinear aspects of the dynamics, such as susceptible feedbacks, are expected, but are hard to model explicitly given complexities in the biology and/or a paucity of data available to reflect these (e.g. multiple strains, asymptomatic carriage). The wavelet spectra allow partitioning of frequencies reflecting such biologically driven resonances (consistent with the intrinsic disease dynamics), allowing other frequencies and their associations with extrinsic variables (e.g. climate variables) to be evaluated. However, the inference remains correlative rather than causative, because climate drivers such as ENSO may have multi-annual cycles that align by chance with multi-annual dynamics of an infectious disease. But if multiple lines of evidence (e.g. average age of infection, exponential growth rates) suggest that the transmission rate of an infection and associated intrinsic dynamics are inconsistent with the observed multi-annual cycles, yet these cycles align with a climatic variable such as ENSO, this provides supporting evidence for a role of this climate variable on transmission.

### (c) Regression-based TSIR models to extract transmission

A potentially powerful approach is to harness statistical methods within a mechanistic framing. One such semi-mechanistic approach examines associations between climate and the *transmission rate* by reconstructing the underlying susceptible and infectious populations. As with Hope Simpson's approach [44], the idea is to condition on susceptibility and exposure to the pathogen in order to extract a measure of ‘infectiousness’, although here the rate of exposure must be inferred from the underlying time series. This concept underlies time-series susceptible–infected–recovered regression models [43], which build on estimating the size of the susceptible population (*S_t_*) by taking into account depletion of susceptibles via infection or vaccination, and replenishment via births. The number of incident infections at time *t* + 1 is then modelled as *I_t+1_* = *β_t_I_t_S_t_*, where *β_t_* is the rate of transmission from an infected to susceptible individual; the time-step should approximately equal the serial interval of the infection. This equation can be linearized by taking the log of both sides, then fitted using a regression with *I_t_* (the observed incidence, corrected for underreporting) and *S_t_* as offsets:

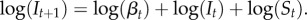

Seasonal (and other temporal) fluctuations in the transmission rate *β_t_* can then be estimated by choosing an appropriate focal time-scale. The resulting estimates of transmission can be analysed to evaluate their association with seasonal climatic (and non-climatic) drivers. Alternatively, *β_t_* can be framed as a function of climatic variables or other intermediate variables (e.g. environmentally sampled viral or bacterial particles) and these associations can be directly evaluated. For pathogens that are not completely immunizing, waning of immunity can be captured (e.g. using Taylor expansions [37]). Such approaches have been used to suggest that seasonal migration patterns linked to agriculture shape measles dynamics in Niger [55], and reveal temperature-dependence of rotavirus transmission in England and The Netherlands [56].

### (d) Partitioning drivers of *R*_E_

A related approach is to extract the time-varying effective reproductive number *R*_E,*t*_ from time series of incidence [57], defined at time *t* as *R*_E*,t*_ = *R*_0_*S_t_*, where *R*_0_ is the constant basic reproductive number (i.e. the average number of secondary infections expected from one infectious individual in a fully susceptible population), and *S_t_* is the number of susceptibles at time *t*; *R*_E*,t*_ is thus the expected number of secondary infections accounting for population immunity. This relationship can be expanded to encompass the influence of potential drivers [31]:

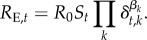

Variables associated with hypothesized mechanisms by which climatic variables shape transmission can be incorporated via the *k* potential drivers, *δ_t,k_*. Following linearization (taking logs, as above), the coefficients *β_k_* can be estimated via regression (e.g. revealing how absolute humidity and school holidays shape influenza-like illness in The Netherlands [31]).

### (e) Dynamic models

The next extension is to fit a full transmission dynamic model to incidence data. Such models explicitly attempt to represent reasonably complex life cycles, and differentiate between infection and disease. Again, there are two possible approaches. First, data from climate drivers can be directly used within the dynamic model. For example, Shaman *et al*. [58] developed a dynamic model for influenza encompassing a parametric relationship between absolute humanity and *R*_0_. A similar approach was used to examine associations between rainfall and cholera incidence in Haiti [59]. Experimental data on the relationship between temperature and mosquito demography were incorporated into a model that revealed how climate shaped the first reported dengue outbreak in Europe [60]; similar models have been used to hindcast seasonal outbreaks of malaria in India to evaluate an early warning system [61]. Full dynamical models can also be framed to allow formal statistical comparisons between hypotheses (e.g. to evaluate the role of rainfall versus feedbacks due to immunity in driving malaria cycles [62]). Alternatively, seasonality in transmission can be approximated (e.g. using a sinusoidal forcing function, for which the amplitude and phase are directly estimated), and association with climate drivers evaluated post-hoc. This approach has been used to examine the climatic drivers underlying the distinct seasonal and spatial patterns of RSV in the USA [35] and the Philippines [63]. Out-of-sample model validation is always essential, the impact of interventions must be considered [64] and care must be taken to avoid common inferential pitfalls (below).

### (f) Risk maps

For vector-borne and zoonotic diseases where endemicity is shaped by the local environment, risk maps estimate the spatial distribution of human risk of infection, capturing heterogeneity at local [65], regional, country-wide [66,67] and global scales [68–71]. Risk-mapping approaches integrate observed occurrences (i.e. prevalence or incidence data) at discrete locations to generate an interpolated map of estimated risk of human infection and/or disease, generally building on environmental, ecological, socio-demographic and/or human susceptibility covariates [69,72], and sometimes known biological dependencies (e.g. of vectors on climate [73]). Underlying methods range from model-based geostatistics (especially where data are rich), to machine-learning approaches (e.g. boosted regression trees [71]), and generally encompass formal quantification of uncertainty [74,75].

Increasingly, methods underlying risk maps encompass core biological mechanisms via transmission models, allowing attribution of the impact of interventions [76] through to projection of burden, for example, by inferring attack rates and modelling demographic processes for Zika virus [77]. Dynamic spatial risk maps based on mechanistic models enable hypothesis-testing of the climatic drivers of infectious disease risk, and attribution of specific events, such as the role of the 2015 El Niño in the emergence of Zika virus [78]. However, parametrizing such risk-map models requires detailed biological and epidemiological knowledge and may be difficult to develop for broad spatio-temporal scales. Model validation against observed changes in the spatial distribution of disease is essential.

## 5. Methodological challenges for climate–disease models

The analysis of links between climate and infectious disease incidence using population-level data raises a series of statistical challenges (beyond the broader epistemological challenges that arise from attempting to indirectly infer mechanism, and process-based challenges; figure 1). First, for any pathogen, there are many possible extractions of climate covariates, which are likely to be highly collinear. In the best-case scenario, clear candidate predictors present themselves through knowledge of biological mechanisms, usually from experimental data (electronic supplementary material, table S1). The most compelling evidence for quantifying the impacts of climate on infectious diseases comes when experimental evidence for the climate–pathogen relationship is used to parametrize models describing population-level patterns at broad spatial and temporal scales (e.g. [79,80]). Where no information is available to guide covariate choice, care should be taken to avoid pitfalls commonly encountered in regression featuring multiple comparisons [81]. Approaches such as principal component analysis and Lasso might be used to reduce the dimensionality of the system, while Bayesian model averaging can be used to identify combinations of potential predictors that provide the best fit to the data while accounting for model uncertainty. If strong relationships between principal components and disease incidence or transmission emerge, the climatic variables captured by the focal principal component can be further explored in laboratory or field experiments to pinpoint potential individual-level mechanisms.

The repeatability of seasonality in both climate variables and disease incidence brings another challenge. For example, a low absolute humidity and low influenza incidence occur at approximately the same time of the year, spurious associations are possible. A permutation test can address this—the time course of drivers within each year is randomly redistributed across years and the analyses re-run [31]. Only parameters whose association is significantly stronger than in the randomized time-series are likely to reflect a true association. Alternatively, associations between the *residuals* of climatic factors and model-predicted incidence can be explored to determine whether *unusual* climatic conditions (that differ from the expected conditions given the time of year) are also associated with higher or lower disease incidence [35,58]. There is promise but also challenge in harnessing the impact of extreme events to strengthen inference [82]. ‘Model-free methods’, such as convergent cross-mapping have been used to infer a role of absolute humidity on influenza [42]; however, the scope of inference with such methods may be limited in settings of periodic fluctuations [83].

A final statistical challenge is the potential lack of congruence between the spatial and temporal scale of available data and the key scale of mechanism [84]. For example, if one day's strong rainfall results in a pulse in transmission of an enteric infection, but disease incidence data are only available monthly, or at a larger spatial scale, this detail will be missed. It has been suggested that this type of mismatch may result in stronger inference with broad-scale trends like El Niño rather than specific climate drivers like temperature [85].

## 6. Methodological challenges related to climate data and climate change models

Observations of atmospheric and hydrological variables will be key to deploying the methods described above. Routine observations are made from surface stations [86], weather balloons [87], satellite radiometers [88], etc. These are used with fluid dynamical constraints to produce estimates of the global atmospheric state at resolutions of tens of kilometres [89]. All of these data sources have uncertainties and biases; consequently, the appropriate source depends on the question of interest, pathogen biology and the spatio-temporal scales of the infectious disease data. Potential variables of interest include directly observed quantities such as temperature or precipitation, or derived measures of heat stress or drought.

Disease incidence in future decades would ideally be predicted using output from climate models, but this effort is hampered by poor climate model resolution and high model uncertainty and bias. Infectious diseases are often sensitive to variability on short spatial scales that is not well-represented by global climate models, with typical horizontal resolutions on the order of 100 km. Statistical or dynamical downscaling—the latter employing higher-resolution climate models integrated only in a particular region—can provide data at the requisite scales, but rely on potentially biased boundary conditions obtained from global models. Global models also typically produce biased estimates of extreme events, such as tropical precipitation extremes [90]; this is problematic as the dynamics of infectious diseases may be more sensitive to extremes or variability than to time-mean properties, and associations may be strongly nonlinear.

However, there is opportunity to identify disease–climate associations that rely on climatic variables with more certain projected changes. For example, although predictions of next-century regional precipitation change are highly uncertain, there is evidence that aridity over many continental regions has increased and will continue to do so in coming decades [91]. The seasonal cycle of rainfall is biased in models of some tropical regions [90], with, for example, potentially serious consequences for malaria burden projections. However, projected delays in the seasonal onset of monsoon rainfall as climate warms are robust across models and stem from well-understood atmospheric thermodynamics [92]. Tight interdisciplinary work, in which climate scientists work closely with infectious disease modellers, thus has the potential to lead to discovery of emergent constraints on future disease incidence.

## 7. Linking climate change models to infectious disease models

Forecasting future infectious disease incidence under climate change requires jointly modelling both changes to future climate and the climate–disease relationship, accounting for uncertainties in both. On short time scales, data-assimilation approaches used in weather forecasting have been applied to influenza [93], taking as a premise noisy data and using statistical filtering to reduce forward error propagation [94] to provide reliable real-time forecasts of influenza incidence across a large number of US cities [93,95], as well as allowing nuanced evaluation of different data-assimilation and filtering approaches [94]. Across longer time scales, an array of possible climate and infectious disease models can be combined in a multimodel ensemble framework, allowing formal assessment of the range of possible future outcomes and identification of the origin of discrepancies between models [96]. This approach has been used to forecast future malaria distributions under climate change—one of the few pathogens for which the necessary diversity of models for the climate–disease relationship exists [80].

For infectious diseases, as for other anticipated impacts of climate change [96], the footprint of human adaptation, ranging from interventions such as vector control to vaccination strategies altering the landscape of immunity, or human movement shifting diseases, complicate forecasting. But near-term forecasts [93] or longer-term projections that explicitly do not account for these subtleties [80] provide a powerful starting point, from which refinements can subsequently be made. In both instances, model projections must be continually tested by comparing with out-of-sample data, and updated when the models fail to capture relevant patterns in data. One potentially fruitful direction is to build on methods used in the climate community to increase skill via retrospective analyses of weather and climate forecast models [93]. Retrospective forecasting of infectious disease outcomes on time scales of months to years would allow the impact of the seasonal cycle, ENSO and other interannual climate variability to be assessed [97]. Importantly, even if the climate prediction itself is wrong, an ensemble approach could assess whether infectious disease projections conditioned on the actual climate occurrence exhibit reasonable skill.

## 8. Conclusion

To evaluate the impact of climate change on the incidence of infectious diseases, we must move beyond identifying simple correlations and statistical associations between climatic variables and incident cases of disease to identifying the underlying causal mechanisms. Just as the methods used for weather forecasting differ from those used for long-range climate projections, different methods are needed to make out-of-sample predictions about future trends in incidence under climate change. Traditional statistical approaches are useful under certain conditions (electronic supplementary material, table S2), for exploratory analyses, and even for short- to medium-term forecasting (e.g. [98]). However, more mechanistic modelling approaches are needed if climatic drivers impact the *transmission* of infection from infected to susceptible hosts. Epidemiologists can take cues from the climate modelling community by seeking to better understand and incorporate the underlying properties that influence the observed behaviour of the climate–disease system, and by routinely testing an ensemble of models by retrospectively comparing and validating models against data. From forecasting influenza epidemics over the short term [94] to disentangling the impact of climate change on malaria [80], great strides are being made, but much remains to be done.

## Supplementary Material

Supplement S1

## Supplementary Material

Table S1

## Supplementary Material

Table S2

## Supplementary Material

Table S3

## Data accessibility

This article has no additional data.
