# Supplementary material for: Identifying climate drivers of infectious disease dynamics: recent advances and challenges ahead
Source: Proc Biol Sci. 2017 Aug 16;284(1860):20170901. doi: 10.1098/rspb.2017.0901 (PMC5563806; doi:10.1098/rspb.2017.0901)
Supplement: Supplement S1 [file rspb20170901supp1.docx]

**Supplement S1: Seasonal fluctuations in incidence and climatic variables: confounders and complications, particularly for directly transmitted infections**

Climate is one of many potential drivers that can shape seasonal fluctuations in pathogen transmission and/or disease incidence. Here, we outline a range of other drivers that might confound inference into seasonal associations between climatic drivers and disease incidence, using the example of directly transmitted infections (for which the implications are more immediate) for illustration.

First, directly transmitted infections require that individuals be in close proximity for transmission to occur. Such infections thus often show a strong dependence on ***human behavior***, which may in some cases lead to seasonal fluctuations in transmission that are not directly driven by climate. For example, ***seasonal aggregation of children in schools*** is a classic driver of the dynamics of directly transmitted childhood infections such as measles [1, 2], and one with little relevance to climate. However, other causes of population aggregation, such as ***seasonal migration linked to agriculture, fisheries, and pastoralism*** [3], have also been suggested to shape measles [4] and meningitis [5] dynamics in sub-Saharan Africa, and likely do change in response to climate. Deployment of ***control efforts*** such as vaccination (or, for vector-borne pathogens, bed-net distribution) is perhaps the aspect of human behavior with the greatest potential to obscure the signal of climate on infectious disease transmission. Control efforts often tend to focus on regions and time periods in which transmission is most intense (e.g., provision of bed-nets may be focused on areas of high transmission and concentrated during the season of greatest mosquito abundance). If this spatiotemporal variation in control efforts is not taking into account, the result will be to flatten out the signal of climate on transmission.

Another important consideration arises for pathogens that are also immunizing (e.g., measles, rubella). ***Human patterns of conception*** are often seasonal [6], resulting in seasonal variation in birth rates, and thus seasonal replenishment of the susceptible population, which could also affect disease dynamics [7, 8]. Whether climate *per s*e or some other signature of periodicity (such as day length) is responsible for seasonality in births remains unclear, but could be an intriguing indirect climate driver of infectious disease dynamics, as well as a potential confounder of inference into direct effects of climate.

An issue that affects directly transmitted pathogens, but also likely affects pathogens more broadly, is the role played by ***interactions between pathogens***. For example, there is evidence that RSV and influenza increase the risk for invasive disease caused by *Streptococcus pneumoniae* [9]; consequently, any apparent climatic signature in the incidence of invasive pneumococcal disease might be an indirect result of the effect of climate on its associated viruses.

Finally, climate may shape the length and intensity of the ‘hunger season’ in regions with high dependence on local agricultural production [3], which may also affect susceptibility to infection; this has been suggested as a possible driver of RSV seasonality in the Philippines, for example [10]. This sort of effect might also play out through immune function (via vitamin D and sunlight effects, for example [11]). If such ***seasonal components of host health status*** are neglected, this could thus be another source of confounding for inference into climate drivers of an infection.

The magnitude of such alternative drivers need not be large to affect seasonal fluctuations in incidence, since the inherent non-linear dynamics associated with infectious diseases can mean that even very small climatic effects on transmission can be amplified, resulting in large observed fluctuations in incidence [12]. Considering the importance of such alternative drivers is thus essential for robust inference into a role of climate for such pathogens.

**References**

1. Metcalf, C.J.E., O.N. Bjørnstad, B.T. Grenfell, and V. Andreasen, *Seasonality and comparative dynamics of six childhood infections in pre-vaccination Copenhagen.* Proceedings of the Royal Society of London, Series B, 2009. **276**: p. 4111-4118

2. Bjørnstad, O.N., B. Finkenstadt, and B.T. Grenfell, *Endemic and epidemic dynamics of measles: Estimating epidemiological scaling with a time series SIR model.* Ecological Monographs, 2002. **72**: p. 169-184.

3. Rain, D., *Eaters of the dry season: circular labor migration in the West African Sahel*. 1999, Oxford: Westview Press.

4. Ferrari, M.J., R.F. Grais, N. Bharti, A.J.K. Conlan, O.N. Bjørnstad, L.J. Wolfson, P.J. Guerin, A. Djibo, and B.T. Grenfell, *The dynamics of measles in sub-Saharan Africa.* Nature, 2008. **451**: p. 679-684.

5. Bharti, N., H. Broutin, R. Grais, M. Ferrari, A. Djibo, A. Tatem, and B. Grenfell, *Spatial dynamics of meningococcal meningitis in Niger: observed patterns in comparison with measles.* Epidemiology and infection, 2012. **140**(08): p. 1356-1365.

6. Currie, J. and H. Schwandt, *Within-mother analysis of seasonal patterns in health at birth.* Proceedings of the National Academy of Sciences, 2013. **110**(30): p. 12265-12270.

7. Martinez-Bakker, M., K.M. Bakker, A.A. King, and P. Rohani, *Human birth seasonality: latitudinal gradient and interplay with childhood disease dynamics.* Proceedings of the Royal Society of London B: Biological Sciences, 2014. **281**(1783): p. 20132438.

8. He, D. and D.J. Earn, *Epidemiological effects of seasonal oscillations in birth rates.* Theoretical population biology, 2007. **72**(2): p. 274-291.

9. Weinberger, D.M., K.P. Klugman, C.A. Steiner, L. Simonsen, and C. Viboud, *Association between Respiratory Syncytial Virus activity and pneumococcal disease in infants: A time series analysis of US hospitalization data.* PLoS Medicine, 2015. **12**(1): p. e1001776.

10. Paynter, S., L. Yakob, E.A. Simoes, M.G. Lucero, V. Tallo, H. Nohynek, R.S. Ware, P. Weinstein, G. Williams, and P.D. Sly, *Using mathematical transmission modelling to investigate drivers of respiratory syncytial virus seasonality in children in the Philippines.* PloS One, 2014. **9**: p. e90094.

11. Stevenson, T., M. Visser, W. Arnold, P. Barrett, S. Biello, A. Dawson, D. Denlinger, D. Dominoni, F. Ebling, S. Elton, N. Evans, H.M. Ferguson, R.G. Foster, M. Hau, D.T. Haydon, D.G. Hazlerigg, P. Heideman, J.G.C. Hopcraft, N.N. Jonsson, N. Kronfeld-Schor, V. Kumar, G.A. Lincoln, R. MacLeod, S.A.M. Martin, M. Martinez-Bakker, R.J. BNelson, T. Reed, J.E. Robinson, D. Rock, W.J. Schwartz, I. Steffan-Dewenter, E. Tauber, S.J. Thackery, C. Umstatter, T. Yoshimura, and B. Helm, *Disrupted seasonal biology impacts health, food security and ecosystems.* Proceedings of the Royal Society B: Biological Sciences, 2015. **282**(1817): p. 20151453.

12. Dushoff, J., J.B. Plotkin, S.A. Levin, and D.J. Earn, *Dynamical resonance can account for seasonality of influenza epidemics.* Proceedings of the National Academy of Sciences, 2004. **101**(48): p. 16915-16916.
