# Supplementary material for: Identifying climate drivers of infectious disease dynamics: recent advances and challenges ahead
Source: Proc Biol Sci. 2017 Aug 16;284(1860):20170901. doi: 10.1098/rspb.2017.0901 (PMC5563806; doi:10.1098/rspb.2017.0901)
Supplement: Table S1 [file rspb20170901supp2.docx]

**Table S1.** Potential mechanisms through which climate can impact the incidence of infectious diseases, organized by mode of transmission and climate mechanism. The ‘Additional evidence needed’ column provides some examples that relate primarily to the focal component in the causal chain linking climate to transmission, but are occasionally more broad-ranging; these are not intended to be comprehensive but provide a range of climate and infectious disease links. See Table S2 for extensions related to climate refugees.

| **Mode of transmission** | **Climate mechanism** | **Example** | **Nature of the evidence** | **Additional evidence needed** | **Sources** |
| --- | --- | --- | --- | --- | --- |
| Airborne | Pathogen survival | Influenza survival and transmission via aerosols varies with absolute humidity. | *In vivo* experiments in guinea pigs and dynamic models fit to incidence of excess pneumonia and influenza mortality at broad temporal and spatial scales across the United States. | Evidence for mechanisms underlying the more complex relationship between absolute humidity and influenza seasonality observed in the tropics. | (3, 99) |
|  | Host defences | Innate immune responses to rhinovirus depend on temperature. | *In vivo* experiments in mice and *in vitro* experiments in human airway cells. | Evidence for increased risk of infection with rhinovirus in cooler temperatures under natural conditions. | (5, 100) |
|  | Host behaviour | Aggregation indoors during cold or rainy season facilitates influenza virus transmission. | Largely theoretical. | Evidence that time spent indoors increases seasonally as hypothesized; evidence that time indoors is associated with increased transmission. | (101) |
| Food- and/or water-borne | Pathogen survival and/or replication | *Vibrio cholerae* growth and attachment to copepods depends on water temperature, pH, and salinity. | *In vitro* laboratory experiments and statistical and semi-mechanistic models showing an association between cholera incidence, phyto-plankton abundance, sea surface temperature and the El Niño-Southern Oscillation (ENSO) | Evidence that *V. cholera* survival in natural transmission settings depends on climate variables; evidence that the strains of *V.* cholera found in aquatic reservoirs precede and are capable of causing human outbreaks of disease; evidence to resolve whether associations with ENSO are mediated through pathogen survival versus human exposure due to increased rainfall. | (38, 102, 103) |
|  | Risk of human exposure | Flooding increases exposure to leptospirosis. | Observational studies. | Evidence that flooding does not also have a suppressive effect on leptospirosis reservoir hosts (e.g. by flooding rat burrows); better understanding of the additional factors that mediate exposure to contaminated environments. | (104) |
| Vector-borne | Vector survival | Survival of adult *Aedes aegypti and Ae. albopictus* mosquitoes is temperature-dependent; *Ae. albopictus* has higher survival than *Ae. aegypti* but lower temperature range tolerance. | Modelling synthesis of 351 laboratory and 59 field survivorship experiments. | Evidence that these patterns are consistent across different vector populations, and that the effect is of a sufficient magnitude to be relevant in the face of other sources of variation. | (105) |
|  | Vector fertility | *Aedes albopictus* gonotrophic cycles (number of days gravid mosquitoes take to oviposit after a bloodmeal) is temperature-dependent | *In vivo* laboratory experiments on an Indian Ocean population | Evidence that this temperature dependence is consistent across populations, and is of a sufficient magnitude to be relevant in the face of other sources of variation. | (106) |
|  | Vector geographic distribution | Expansion of *Ixodes ricinus* northern latitude limit in Sweden correlated with warmer winters | Field studies on *I. ricinus* distribution and density across Sweden.. | Evidence for underlying mechanistic drivers (i.e., experiments showing that vector survival and vector dispersal dependence on climate). | (107) |
|  | Vector behaviour | *Ixodes scapularis* phenology (feeding activity) is shifted earlier in warmer years. | Field studies on *I. scapularis* burdens on small mammal hosts in New York. | Evidence that seasonal synchrony in larval and nymphal ticks is robust and overwhelms effects of day length, and thus might alter pathogen prevalence as the climate shifts. | (108) |
|  | Vector development rate | *Ixodes scapularis* developmental periods decreased with increasing temperature. | *In vivo* laboratory and field experiments on Canadian tick populations. | Evidence that *I. scapularis* developmental periods are influenced by temperature (as well as day length) in natural settings, and across their range. | (109) |
|  | Pathogen development rate (extrinsic incubation period) | Temperature mean as well as daily temperature fluctuations influence *Plasmodium falciparum* development within the Asian malaria vector. | *In vivo* laboratory experiments infecting the Asian malaria vector *Anopheles stephensi* with rodent malaria *Plasmodium chabaudi.* | Evidence that this effect is of sufficient magnitude to drive outcomes in natural settings. | (16) |
|  | Vector susceptibility to infection | Chikungunya virus (CHIKV) infection, dissemination, and viral titer in Aedes albopictus depend on larval temperature | *In vivo* laboratory experiments on  Florida F_1_ population of Ae. albopictus | Evidence that larval *and* adult temperatures affect vector susceptibility in natural settings. | (110) |
|  | Risk of human exposure | Children are less likely to sleep under permethrin-treated bed nets during warmer weather. | Randomized controlled trial of factors affecting use of bed nets in western Kenya. | Evidence for the importance of this effect in the face of other human behavioural changes that might affect mosquito exposures over the time-course of climate change. | (111) |
| Additional considerations for zoonotic pathogens | Geographic range of reservoir species | Areas of plague activity in California are temperature-dependent and predicted to shift under climate change. | Ecological niche modelling of plague foci from surveillance of California ground squirrel plague hosts. | Evidence of the magnitude of the contribution of rodent host range shifts on plague distribution. | (112) |
|  | Population dynamics of reservoir species | Climatic effects on deer mouse population dynamics influence risk of human exposure to hantavirus. | Capture–mark–recapture study and population dynamics modeling of deer mouse populations over 15 years in Montana. | Evidence of common drivers of deer mouse population dynamics in the southwestern US, for example, a region with distinct climate and habitat. | (113) |
|  | Reservoir species behaviour | Climate influences migration timing and avian influenza virus prevalence in a wild bird reservoir host | Mathematical model of ruddy turnstone bird population dynamics and avian influenza virus prevalence in Delaware Bay. | Evidence for a role of migratory birds in long-distance dispersal of avian influenza viruses. | (114, 115) |
