# Supplementary material for: Identifying climate drivers of infectious disease dynamics: recent advances and challenges ahead
Source: Proc Biol Sci. 2017 Aug 16;284(1860):20170901. doi: 10.1098/rspb.2017.0901 (PMC5563806; doi:10.1098/rspb.2017.0901)
Supplement: Table S2 [file rspb20170901supp3.docx]

**Table S2: Guidelines for applying different analytical methods to examine associations between climatic variables and disease incidence or prevalence.**

| **Questions to consider** | **Statistical modeling approaches**  *e.g. Generalized linear models, ARIMA models, wavelet analysis* | **Mechanistic and semi-mechanistic modeling approaches**  *e.g. TSIR models, Estimation of time-varying reproductive numbers, dynamic models* |
| --- | --- | --- |
| Are there *hypothesized mechanisms* through which climate may affect disease risk? | No, exploratory analyses are needed to identify associations between climate and infectious disease incidence.  Example: Autoregressive integrated moving average (ARIMA) models have been used to forecast the incidence of cutaneous leishmaniasis in Brazil, and identified significant inverse associations with relative humidity and precipitation at 3-5 month lags (1) | Yes, analyses should focus on identifying and quantifying the causal mechanisms underlying the hypothesized associations.  Example: The effect of temperature on vector dynamics was incorporated into a dynamic model of dengue transmission to demonstrate the role of climate in shaping an outbreak in Portugal, and evaluate the risk of future outbreaks and establishment of endemic transmission (2). |
| Is the effect of climate mediated through the *observation process* or *transmission process*? | The effect of climatic variables is on the *observation process* (e.g. the probability of a colonizing bacteria causing invasive disease) rather than the transmission process.  Example: Poisson regression was used to model the incidence of invasive group A streptococcal infections, and demonstrated that both incidence and severity peaked in January-April in Iceland; associations with specific climatic factors have not been examined (3). | The effect of climatic variables impacts the *incidence AND transmission* of disease (e.g. cases of disease are more infectious than subclinical infections).  Example: A dynamic model for RSV transmission was fit to time series data from different states across the US; seasonal variation in the transmission rate was correlated post-hoc with variability in climatic parameters, and significant positive associations with potential evapotranspiration were identified (4). |
| If there is a vector / reservoir host in the life-cycle, is the pathogen vector/reservoir host undergoing *range expansion or changes in geographic distribution*? | No, the vector/host range is static over epidemiological timescales.  Example: Statistical pattern-matching approaches were used to examine correlations between the spatial distribution of tick abundance, reported tick-borne encephalitis virus (TBEv) infections, and seasonal profiles of temperature and vegetation indices across seven European countries; rapid decreases in land surface temperature in autumn were associated with larval-nymphal synchrony and TBEv presence/absence (5). | Yes, the vector/host may undergo rapid range changes that underlie human pathogen exposures.  Example: An agent-based dynamic model was used to project the impact of climate change on the geographic distribution of *Schistosoma mansoni* in eastern Africa, accounting for non-linear effects of water temperature on schistosome transmission (6). |
| If there is a zoonotic reservoir, does it go through *boom/bust cycles related to climate*? | No, climate ONLY affects the risk of human exposure to a zoonotic pathogen (e.g., for livestock based pathogens).  Example: A negative binomial regression model was used to evaluate the effect of temperature on the risk of food-borne salmonellosis originating in livestock in Australia; higher ambient temperature in the previous month was associated with the incidence of salmonellosis cases (7). | Yes, climate affects the population dynamics of zoonotic reservoir species with complex non-linear feedbacks.  Example: A discrete-time model was used to simulate the effects of temperature and rainfall on deer mouse population dynamics, which modulates the risk of hantavirus pulmonary syndrome in humans (8); density dependent feedbacks in the host population require dynamic modeling. |
| Does infection lead to some form of *immunity* against reinfection and/or risk of disease given infection? | Infection is non-immunizing.  Example: Generalized linear models were used to evaluate the role of climatic and environmental variables in the spatial distribution of hookworm in South Africa; minimum temperature and number of rainy days in January were predictive of prevalence (9), with higher prevalence in locations with higher temperatures. | Immune feedbacks modulate the possible effects of climate on disease incidence.  Example: A modified TSIR model was used to analyze associations between climate and cholera incidence in Bangladesh over a 36-year period; correlations between cholera transmission and monsoon rains, sea surface temperature in the Bay of Bengal, and ENSO were identified after controlling for population immunity (10). |
| If the infection is immunizing, what is the magnitude of the basic reproductive number (*R*_0_) and generation interval? | Pathogens with a low *R*_0_ and long generation interval, since the non-linear feedbacks leading to dependence among consecutive observations of disease incidence or prevalence will be weak (and therefore potentially can be ignored) at short timescales.  Example: Wavelet analysis was used to examine associations between meningitis disease incidence and climatic variables and measures of atmospheric dust in Niger; significant coherency was observed between meningitis incidence and dust in all study years and across all districts (11). | Acute pathogens for which the prevalence of infection (and therefore incidence of disease) can change on similar timescales to the climatic drivers of interest.  Example: Weekly effective reproductive numbers (*R_E,t_*) were estimated based on a 41-year time series of influenza-like illness incidence in the Netherlands, and associations between *R_E,t_* and absolute humidity, as well as school holidays; absolute humidity explained 3% of the variance in weekly influenza reproductive numbers (12). |

**References**

1. Lewnard JA, Jirmanus L, Júnior NN, Machado PR, Glesby MJ, Ko AI, et al. Forecasting Temporal Dynamics of Cutaneous Leishmaniasis in Northeast Brazil. PloS Neglected Tropical Diseases 2014;8:e3283.

2. Lourenço J, Recker M. The 2012 Madeira dengue outbreak: epidemiological determinants and future epidemic potential. PLoS Negl Trop Dis. 2014;8(8):e3083.

3. Olafsdottir L, Erlendsdóttir H, Melo-Cristino J, Weinberger D, Ramirez M, Kristinsson K, et al. Invasive infections due to *Streptococcus pyogenes*: seasonal variation of severity and clinical characteristics, Iceland, 1975 to 2012. Euro Surveill. 2014;19(17):5-14.

4. Pitzer VE, Viboud C, Alonso WJ, Wilcox T, Metcalf CJ, Steiner CA, et al. Environmental drivers of the spatiotemporal dynamics of respiratory syncytial virus in the United States. PLoS Pathogens. 2015;11(1):e1004591.

5. Randolph S, Green R, Peacey M, Rogers D. Seasonal synchrony: the key to tick-borne encephalitis foci identified by satellite data. Parasitology. 2000;121(01):15-23.

6. McCreesh N, Nikulin G, Booth M. Predicting the effects of climate change on Schistosoma mansoni transmission in eastern Africa. Parasites & vectors. 2015;8(1):1.

7. D’Souza RM, Becker NG, Hall G, Moodie KB. Does ambient temperature affect foodborne disease? Epidemiology. 2004;15(1):86-92.

8. Luis AD, Douglass RJ, Mills JN, Bjørnstad ON. The effect of seasonality, density and climate on the population dynamics of Montana deer mice, important reservoir hosts for Sin Nombre hantavirus. Journal of Animal Ecology. 2010;79(2):462-70.

9. Mabaso M, Appleton C, Hughes J, Gouws E. The effect of soil type and climate on hookworm (Necator americanus) distribution in KwaZulu‐Natal, South Africa. Tropical Medicine & International Health. 2003;8(8):722-7.

10. Koelle K, Rodo X, Pascual M, Yunus M, Mostafa G. Refractory periods to climate forcing in cholera dynamics. Nature. 2005;436:696-700.

11. Agier L, Deroubaix A, Martiny N, Yaka P, Djibo A, Broutin H. Seasonality of meningitis in Africa and climate forcing: aerosols stand out. Journal of the Royal Society Interface. 2013;10(79):20120814.

12. te Beest DE, van Boven M, Hooiveld M, van den Dool C, Wallinga J. Driving factors of influenza transmission in the Netherlands. American Journal of Epidemiology. 2013;178:1469-77.
