# Supplementary material for: Identifying climate drivers of infectious disease dynamics: recent advances and challenges ahead
Source: Proc Biol Sci. 2017 Aug 16;284(1860):20170901. doi: 10.1098/rspb.2017.0901 (PMC5563806; doi:10.1098/rspb.2017.0901)
Supplement: Table S3 [file rspb20170901supp4.docx]

**Table S3.** Extension of Table S1 on potential mechanisms through which climate can impact the incidence of infectious diseases: additional considerations related to climate refugees

|  | **Climate mechanism** | **Example** | **Nature of the evidence** | **Additional evidence needed** | **Sources** |
| --- | --- | --- | --- | --- | --- |
| Additional considerations related to climate refugees | Aggregation and displacement of climate refugees | Climate refugees will likely have to live in cramped quarters, be exposed to unsanitary camp conditions, and may be exposed to pathogens for which they have no baseline immunity | Largely theoretical; based on observations from non-climate-related refugee crises | Evidence on disease burden accounting for imperfect surveillance of environmental refugee populations. | P. Martens, L. Hall, Malaria on the move: human population movement and malaria transmission. *Emerging infectious diseases* 6, 103 (2000). |
|  | Climate-induced land-use changes | Changes in land-use related to climate change and refugee movements could shift vector habitats | Largely theoretical | Evidence of *how* land-use will change under different climate regimes, and the relative importance of climate change versus other factors. |  |
